# The Moderating Effect of Community Environment on the Association Between Social Support and Chinese Older Adults' Health: An Empirical Analysis Study

**DOI:** 10.3389/fpubh.2022.855310

**Published:** 2022-04-27

**Authors:** Dongfang Li, Xiaolin Li, Yi Zeng

**Affiliations:** ^1^School of Public Administration, Zhongnan University of Economics and Law, Wuhan, China; ^2^Center for Social Security Studies of Wuhan University, Wuhan University, Wuhan, China

**Keywords:** social support, community environment, older adults, health, mental health

## Abstract

**Background:**

The aging population conundrum and the gradual weakening of older adults' health and ability to obtain resources as they age have drawn attention to this population's health. Older adults' health relates not only to their own quality of life, but also to the development of families/society.

**Methods:**

We analyzed micro data from the 2011 and 2015 waves of the China Health and Retirement Longitudinal Study Follow-up Questionnaire, using the probit model, ordinary least squares model, and other methods.

**Results and Conclusions:**

Both formal and informal social support significantly impacted the physical and mental health of Chinese older adults, and the community environment moderated this relationship. To build a reasonable and effective social support system for older adults and improve their health, we suggest that stakeholders should continue to strengthen the formal and informal social support provided to older adults; they should also build a community-based care system, which will allow for the moderating role of community environment on the relationship between social support and older adults' health. Family and social support factors are important for older adults' health. We should enable the moderating role of community environment on the relationship between social support and health to be fully exerted, as well as build a community-based pension system.

## Introduction and Literature

According to the Seventh National Population Census from National Bureau of Statistics of China, the number of people aged 60 and over was 264.02 million (i.e., 18.7% of the total population), and of people aged 65 and over was 190.64 million (i.e., 13.5%) in 2020 (1). Compared with the Sixth National Population Census in 2010, the proportion of people aged 60 and over increased by 5.44% and the proportion of people aged 65 and over increased by 4.63%. Despite the increase in the older adult population, their health status is not optimistic. The health of the older-adult population is important both for their quality of life and to ensure the stable development of families and society.

The “healthy China” strategy was devised to ensure a “healthy aging” process nationwide, including the construction of a health service system, strengthened health education, and implementation of comprehensive interventions for common and chronic diseases, and mental health and care services for older adults. This strategy underpins how China has given due attention to the health problems of older adults in the country.

Among the factors affecting older adults' health, social support has been playing an increasing role. Older adults often receive economical, emotional, and other types of support from their own social networks. Formal social support (e.g., pensions and medical insurance) provides direct economic support, ensuring their medical care and the daily care of family members. Meanwhile, informal social support (e.g., spiritual comfort) has an important impact on their mental health. Additionally, factors such as community environment, relationship between one's family and the community, and the ability to access/obtain community resources impact their physical health status, psychological perception, and social adaptability, which all influence older adults' health. This suggests that social support, community environment, and older adult health may be closely linked. Further, social support may often indirectly influence older adults' health through the community environment, suggesting that different community environments modulate the potential influence that social support has on older adult health.

Many scholars have divided older adult health into physical and mental health (2–5). Self-rated health, number of chronic diseases, and self-care ability have all been found to be common ways of measuring physical health status in older adults (6–8). Mental health status has been measured using constructs including cognition, depression suppression, and interpersonal relationships (9–14).

Most current research on the factors influencing older adults' health has focused on individual characteristics, health awareness, environmental factors, and social support (15–18). That which has focused on social support has mostly analyzed the influence of formal and informal social support. Various types of formal social support have been shown to significantly affect health, such as issuing pensions, purchasing medical insurance or accessing insurance systems, and reimbursement of hospitalization expenses. Specifically, Xu and Liu (19) used difference-in-difference based on the propensity score matching method to find that the New Cooperative Medical Scheme could significantly improve self-care ability and reduce the number of chronic diseases in Chinese older adults. Wu and Jia (20) indicated that formal social support (e.g., basic old-age insurance for urban employees in China, and retirement benefits of government agencies and institutions) can promote health self-assessment. Moreover, Leng and Zhang (21) believed that implementing specific policies, such as the “new rural cooperative medical system” and the “new rural insurance” weakened “family pension” (which they deemed of an irreplaceable nature) to a certain extent, and significantly impacted older adults' daily life, especially their physical and mental health. Li (22) and Liu (23) reached similar conclusions.

Regarding informal social support, some factors affecting older adults' health are economic assistance, provision of daily care, and spiritual comfort from their children, siblings, and/or other family members. For instance, Wang and Li (24) demonstrated that home care can reduce the degree and the possibility of depression in older adults with disabilities. Sun and Ji (25) indicated that intergenerational support is an important factor affecting older adults' mental health, with adult children's provision of financial and housework support having a positive effect on mental health. Further, Ao (26), using data from a rural sample from the China Rural Urban Migration Survey, found that rural left-behind older adults' health is significantly negatively impacted by their children's abandonment. However, Kuhn et al. (27) demonstrated that children's act of going out to work increases financial support for the parents, having a positive impact on parental health.

Regarding community environment, many studies described that community infrastructure, activity places, and medical institutions can affect older adults' health to varying degrees (28–31). Other studies have analyzed community environment as a moderator of the relationship between different factors and older adults' health. For example, Peng (32) indicated that the “richness of health facilities” negatively moderates income's impact on health, which is to say that by increasing such richness, the health inequality evoked by income factors can be effectively reduced. Zheng (33) also wrote that community sports facilities moderated the impact of income, education, age, and household registration on health; specifically, the infrastructure of community sports facilities was conducive to improving the depression level of middle-aged and older adults with low income, low education, and rural household registration. Jin et al. (34) demonstrated that community environment moderates the impact of living arrangements on older adults' depression tendency. Particularly in communities with few cultural activities, this tendency was the lowest among older adults living with their children and highest among those living alone; in communities with rich cultural activities, there was little difference in depression tendency among these two groups.

In summary, the literature has generally unilaterally evaluated self-rated physical health or mental health, lacking comprehensive research measuring health using both these constructs. Further, although it has been recognized that social support has a significant impact on older adults' health, some topics remain worthy of further discussion, such as the impact of different types of social support and the moderating role of community environment in this relationship. At the same time, most studies conducted in China were limited to a certain region (city or community) or specific groups (neglected older adults, living alone, with disabilities, etc.); they had sample-related methodological limitations, sparking the need to evaluate the representativeness and generalizability of their findings.

Accordingly, we analyzed a national, large-scale data set with information on the health and pensions of older Chinese adults. In order to empirically and comprehensively test the relationship among social support, community environment, and older adults' health, we divided social support into formal and informal types and divided health into physical (i.e., self-rated health and the number of chronic diseases) and mental (i.e., depression and cognitive level) forms. We hope that this research provides stakeholders with an empirical basis to assist building reasonable and effective social-support schemes for older adults against the background of the notions of active aging and healthy aging.

## Materials and Methods

This study was based on microdata from the China Health and Retirement Longitudinal Study Follow-up Questionnaire, conducted in Peking University (hereinafter referred to as CHARLS data). The questionnaire data comprising basic family characteristics, child characteristics, health status, income, and expenditure are all integrated. We tested social support's impact on physical and mental health in different community environments on older adults over 60 years of age. We used data from the 2011 and 2015 waves of the CHARLS. We combined the variables in different databases according to the sample ID, removed the samples that do not meet the age conditions, and deleted the samples with too many missing values. The total number of samples in the original CHARLS database was about 20,936. After screening and processing the CHARLS data from the 2015 wave, we obtained an effective sample size of 10,436 older adults.

## Research Variables

### Explained Variable

We used older adults' health as the Explained variable, dividing it into physical and mental health. Physical health was measured using self-rated health (35) and the number of chronic diseases (36, 37). In the CHARLS, self-rated health was assessed using one question, “What do you think of your health?” Participants chose “Very good, Good, Fair, Poor, Very poor.” To facilitate data analysis, we assigned values to these answers, from high to low (Very good = 5, Good = 4, Fair = 3, Poor = 2, and Very poor = 1). To measure the number of chronic diseases, the CHARLS asked respondents to provide a numerical value within the range of 0–14. The chronic diseases in the CHARLS data include Hypertension, Dyslipidemia, Diabetes, Cancer, Chronic lung diseases, Liver disease, Heart disease, Stroke, Kidney disease, Stomach disease, Psychiatric problems, Memory-related disease, Arthritis, Asthma. Importantly, the CHARLS data contains 14 chronic diseases, and such a large number made reporting the data analysis somewhat complex. Therefore, for the sake of this study, we reported only the proportions from older adults without chronic diseases, with one, and with two or more kinds of chronic diseases.

Mental health was measured using degree of depression (11, 38) and cognitive level (39). In the CHARLS, depression was measured using 10 items about the feelings and behaviors of older adults for the past week (e.g., “I was bothered by things that don't usually bother me” “I had trouble keeping my mind on what I was doing”). The items are answered on a 4-item scale: Rarely or none of the time (<1 day), Some or a little of the time (1–2 days), Sometimes or half of the time (3–4 days), and most of the time (5–7 days); total scores ranged from 10 to 40. To facilitate data analysis and referring to Gao et al. (40) and Xu (11), for positive questions, the higher the score, the higher the degree of positiveness. Negative questions were reverse scored. Regarding total scores, the lower the score, the lower the degree of depression and the better the mental health status. After assessing participants' total scores, we re-coded the data, assigning participants to two groups based on their scores: 1 (no depression) for those with a score of 20 and below, and 0 (depression) for those with scores over 20.

In the CHARLS, cognitive level was measured by a Mini-Mental State Examination form; specifically, participants responded to items on year, month, day, week, and season. One point was assigned to each correct answer, total scores ranged from 0 to 5; the higher the score, the better the cognitive function.

### Explanatory Variables

We used formal and informal social support as the explanatory variable in this study. Formal social support was measured by the indicators of medical insurance (i.e., social medical insurance and commercial medical insurance), old-age insurance (i.e., social old-age insurance and commercial old-age insurance), and other social assistance programs (e.g., minimum living security funds, unemployment endowment, old-age endowment for older adults, etc.). Informal social support was measured mainly by assessing the number of living children, siblings, and financial support received from parents and children.

### Control Variables

To ensure the robustness of the research model, age, sex, marital status, and residency (divided by urban and rural areas) were used as the control variables. The “sex” is considered a categorical variable which is classified as “1 male” and “0 female.” The “marital status” is classified as “married, separated, divorced, widowed, cohabitated, and single.”

### Adjusting Variables

According to the literature review, community environment may moderate the relationship between social support and older adults' health. Part of the 2011 CHARLS assessed community variables, but that section was not included in the data from 2013 and 2015. Based on a study by Zhang and Li (41) and the available CHARLS data, community environment was divided into infrastructure, activity places, and medical institutions indexes.

Community infrastructure included roads, heating system, sewer system, waste treatment system, public toilets, and water and toilet sanitation systems. Community activity places included public facilities, community facilities (e.g., activity centers for older adults), nursing homes, supermarkets, kindergartens, primary and middle schools, etc. Community medical institutions included those that could be visited by community residents, such as community health service centers. The sum of these three indexes yielded the community environment (total) index. We defined a community environment index of <11 (with 11 points being the median) as an average community environment and an index >11 as a good community environment; these classifications were used for conducting sub-sample descriptive statistics and for the regression models using the adjusting variables.

## Model Design

### Ordered Probit Regression Model

The model was as follows:


$$\begin{array}{l} \frac{P\left(\text{self-}reportedhealth\leq j\right)}{1-P\left(\text{self-}reportedhealth\leq j\right)}\;=\;\alpha_{j}\;+\;\beta_{11}x_{11}\;+\;\beta_{12}x_{12}\\ \;+\;\ldots \;+\;\beta_{n}x_{n} \end{array}$$

where self-reported health refers to older adults' self-reported health which is an ordered variable, “*j*” represents the value of self-rated health which is ranging from 1 to 5, “*aj*” represents the constant term of the model and *x*_11_, *x*_12_ … *x*_*n*_ represent the independent and control variables, including social support, sex, age, marital status, and residency.

### Linear Regression Model

The model was as follows:


$$\begin{array}{lll} Num.disease\; & = & \;\beta_{21}socialsupport\;+\;\beta_{22}X\;+\;\varepsilon \\ cognition\; & = & \;\beta_{31}socialsupport\;+\;\beta_{32}X\;+\;\varepsilon \end{array}$$

where *Num.disease* indicates the number of chronic diseases, and cognition refers to the cognitive level, with both being continuous variables. Further, the *X* includes a matrix of variables such as gender, age, marital status, and place of residence. The ε is a residual term.

### Binary Probit Regression Model

The model was as follows:


$$\begin{array}{l} \text{Probit}\left(depression=1\right)\;=\;a_{j}\;+\;\beta_{41}x_{1}\;+\;\beta_{42}x_{2}\;+\;\ldots +\beta_{4n}x_{n} \end{array}$$

where a value of 1 for depression indicates no depression, and the *x*_1_, *x*_2_ … *x*_*n*_ represents the independent and the control variables, including social support, sex, age, marital status, and residency.

## Discussion

### Descriptive Statistics

The total number of samples in the original CHARLS database was about 20,936. We took the elderly group aged 60 and above as the research object, so we combined the variables in different databases according to the sample ID, removed the samples that do not meet the age conditions, and deleted the samples with too many missing values. After screening and processing the CHARLS data from the 2015 wave, we obtained an effective sample size of 10,436 older adults. Although the total sample size was 10,436, there were still missing values for some variables in many participants' data; thus, the sample size for some variables in the econometric and statistical analyses below could be 9,785.

Regarding sex, 4,973 were male (47.7%), with a male-female ratio of 1:1.1; that is, the sample was balanced regarding sex distribution.

Regarding residency, there were 2,918 urban older adults (28%), and the rest were rural; hence, there was a large difference in the distribution of residency.

Regarding informal social support, it has been shown that parental financial support tends to play an important role in health. However, most older adults no longer have living parents; further, those who do are not likely to receive support from them, owing to their advanced age.

### Benchmark Regression

#### Impact of Formal Social Support

Self-rated health, the number of chronic diseases, the degree of depression, and cognitive level were the indexes used in the regression for the impact of formal social support on health (Table 1). After controlling various variables, we achieved the results in columns (1) and (3) for the mean marginal change of each variable in the probit model; columns (2) and (4) show the results of the linear regression model.

**Table 1:** Impact of formal social support on the health of older adults.

	**(1)**	**(2)**	**(3)**	**(4)**
	**Self-rated health**	**Number of chronic diseases**	**Degree of depression**	**Cognitive level**
Endowment insurance	0.005**	−0.024*	0.101***	0.079***
	(0.002)	(0.010)	(0.019)	(0.016)
Medical insurance	−0.020***	0.137***	0.072**	0.188***
	(0.002)	(0.036)	(0.033)	(0.027)
Other social assistance	0.026***	−0.128***	−0.625***	−0.890***
	(0.0035)	(0.0277)	(0.025)	(0.0218)
Sex	−0.208***	−0.172***	0.382***	0.345***
	(0.022)	(0.020)	(0.019)	(0.015)
Age	0.012***	0.044***	−0.006***	−0.018***
	(0.001)	(0.001)	(0.0009)	(0.001)
Urban	−0.219***	−0.038*	0.184***	0.525***
	(0.023)	(0.021)	(0.020)	(0.018)
Marriage	−0.745***	−0.055	4.191	5.475
	(0.148)	(0.116)	(86.28)	(98.45)
Separated	−0.449*	−0.144	3.660	5.114
	(0.255)	(0.226)	(86.28)	(98.45)
Divorced	−0.457**	0.057	3.878	5.366
	(0.183)	(0.154)	(86.28)	(98.45)
Widowed	−0.711***	−0.196	3.999	5.363
	(0.152)	(0.120)	(86.28)	(98.45)
Cohabiting	−0.812**	−0.113	3.632	5.270
	(0.402)	(0.329)	(86.28)	(98.45)
Unmarried (reference group)				
_cons		−1.268***	−3.826	3.504***
		(0.135)	(86.28)	(0.145)
cut1	−1.370***			
	(0.166)			
cut2	−0.885***			
	(0.166)			
cut3	0.495***			
	(0.166)			
cut4	1.446***			
	(0.166)			
*N*	4,845	9,785	9,785	9,785
*R* ^2^	0.013	0.092	0.055	0.204

* *p <0.1,*

** *p <0.05, and*

*** *p <0.01. _cons, constant term; cut, quantile. The number in () means the standard deviation*.

Column (1) shows that older adults with endowment insurance had better self-rated health status. Pensions may increase retired older adults' disposable income, resulting in increased security that may promote positive self-rated health. Older adults with medical insurance might have showed poor self-rated health owing to the “selection effect” of medical insurance; that is, as Cutler et al. (42) described, older adults who buy medical insurance may be more likely to be highly risk averse and consider themselves in a poor health state. The promotion effect of formal social support on self-rated health was also generally found in the results for old-age insurance, and the reasons for this promotion effect seem to be similar to those depicted thus far.

The self-rated health status of females was better than that of males; this may be due to the greater work pressure borne by men. There was also significant positive relationship between age and self-rated health, which may be because they have access to more social insurance benefits, social assistance funds, and have no work pressure as age increases (43, 44). Moreover, rural older adults showed better self-rated health than their urban counterparts; this may be due to lower environmental pollution in rural areas, to rural adults being engaged in agricultural activities throughout the whole year, and to having a good physical status. Furthermore, being married, separated, divorced, widowed, and in cohabitation all had significant negative effects on self-rated health; this may be due to different forms of companionship and the care received from spouses in different marital statuses.

Column (2) shows that endowment insurance and other social assistance programs reduced the number of chronic diseases, while medical insurance increased this number. The results show that access to endowment insurance and other social assistance programs may improve older adults' mental health, which might be due to either the financial support that these sources provide, or to other types of support that they enable. This may make older adults more willing to engage in health activities and reduce the probability of suffering from chronic diseases. Meanwhile, the greater number of chronic diseases among those who had medical insurance may be related to a reduction in health behaviors after obtaining this type of insurance; this may indirectly lead to a higher probability of suffering from chronic diseases. After adjusting for covariates, the number of chronic diseases in males was lower than in females. Further, there was a positive and significant relationship between age and the number of chronic diseases; specifically, with the increase of age, physical function tends to deteriorate, and the number of chronic diseases may tend to increase. The number of chronic diseases was lower in urban older adults than rural; this may be because the healthcare in urban areas is more convenient and higher quality. Being married, separated, divorced, widowed, and in cohabitation had no significant impact on the number of chronic diseases.

Column (3) shows that endowment insurance, medical insurance, and other social assistance programs all significantly impacted the degree of depression; particularly, endowment insurance and medical insurance had a positive impact, while other social assistance programs had a negative impact. The reason might be that, although endowment insurance and medical insurance can afford a greater sense of security owing to greater certainty of receiving such support, the financial support received from other social assistance programs cannot provide such feelings, mostly because they are attached to free assistance initiatives, which are not always consistent; this reality and uncertainty may foster a certain psychological burden in older adults. Furthermore, age and urban residency showed significant effects on the degree of depression: age showed a negative effect, and urban residency a positive effect. The age-related result may be related to older people having a higher tendency toward depression; specifically, as older adults age, they may face greater psychological pressure owing to the fear of death, and thus develop depression. For urban residency, it may be that older urban adults not only have common physical health factors, but also have less social and economic pressure. Further, being married, separated, divorced, widowed, and in cohabitation had positive effects on the degree of depression, albeit not to a significant extent in the regression.

Column (4) shows that endowment insurance, medical insurance, and other social assistance programs significantly affected cognitive levels. Endowment insurance and medical insurance had a positive effect, while other social assistance programs had a negative effect. The reasons for this are similar to those depicted for the previous columns. Regarding the covariates, sex, age, and residency showed significant effects on cognitive level. Regarding sex, the males' cognitive levels were higher than the females; this may be because the males' education level may be generally higher than that of the females, having an impact on their cognitive level (45–47). Regarding residency, the cognitive level of urban older adults was better than that of rural older adults; this may be due to differences in education level, with urban residents generally having higher education levels. Regarding age, those who were older showed a poorer cognitive level, which may be related to advanced-age adults having less knowledge of mental diseases; this has been shown to be detrimental to higher cognition. Additionally, being married, separated, divorced, widowed, and in cohabitation all had positive effects on cognitive level, albeit not to a significant extent in this regression.

The result could be supported by the research of “Lu et al. (48).”

#### Impact of Informal Social Support

We used the same indexes as in the former regression for analyzing the impact of informal social support (Table 2). Formal social support, age, sex, marital status, and residency (divided by urban and rural areas) were used as control variables in the current analysis.

**Table 2:** Impact of informal social support on the health of older adults.

	**(1)**	**(2)**	**(3)**	**(4)**
	**Self-rated health**	**Number of chronic diseases**	**Degree of depression**	**Cognitive level**
Number of surviving children	0.045***	0.041***	−0.046***	−0.030**
	(0.011)	(0.010)	(0.009)	(0.014)
Number of siblings	0.005	0.031***	0.009*	0.053***
	(0.007)	(0.006)	(0.005)	(0.009)
Parental financial support	−0.005	−0.013	−0.003	−0.005
	(0.009)	(0.010)	(0.009)	(0.013)
Children's financial support	0.005***	−0.002**	0.003**	0.005*
	(0.001)	(0.001)	(0.001)	(0.003)
**Control variables**	**Control**	**Control**	**Control**	**Control**
_cons		−1.360***	−0.335**	1.488***
		(0.227)	(0.141)	(0.189)
cut1	−1.487***			
	(0.185)			
cut2	−0.974***			
	(0.184)			
cut3	0.351*			
	(0.184)			
cut4	1.343***			
	(0.185)			
*N*	4,845	9,785	9,785	9,785
*R^2^*	0.125	0.089	0.058	0.222

* *p <0.1,*

** *p <0.05, and*

*** *p <0.01. _cons, constant term; cut, quantile. The number in () means the standard deviation*.

Column (1) in Table 2 shows that the number of living children and children's financial support significantly and positively impact self-rated health; namely, the more living children, the stronger the parents' sense of security and the better their self-rated health. This may be because receiving higher financial support from their living children may directly improve their standard of living, also improving their self-rated health. The number of siblings and parental financial support had no significant effect on self-rated health.

Column (2) shows that the number of living children and number of siblings significantly positively impacted the number of chronic diseases; this may be because the greater the number of children or siblings, the more chores the older adults have to manage, the more help they may have to give, and the more they may need to share, all of which may contribute to developing a chronic disease. Children's financial support had a significant negative impact on the number of chronic diseases; this may be because the financial support can directly improve parents' quality of life and increase their health investment. The effect of parental financial support on the number of chronic diseases was not significant.

Columns (3) and (4) show that the higher the number of living children, the more likely older adults are to experience depression and lower cognitive levels. This may be because a higher number of children increase the likelihood that older adults still have to actively work. Meanwhile, the higher the number of siblings, the less likely older adults were to experience depression, and the better their cognitive levels; this may be because a higher number of siblings increases the likelihood for older adults to share hard work. Furthermore, children's financial support had a significant positive impact on mental health; this may be because such financial support directly improves the quality of life of older adults. The effect of parental financial support on the degree of depression and cognitive levels was not significant.

The result could be supported by the research of “Li and Ge (49).”

### Moderating Effect of Community Environment

To explore the moderating effect of community environment, we conducted an analysis using the three indexes of infrastructure, activity places, and medical institutions. The results of the regression are shown in Tables 3–6.

**Table 3:** Impact of formal social support on physical health of older adults (regulatory effect of community environment).

	**(1)**	**(3)**	**(3)**	**(4)**	**(5)**	**(6)**
	**Self-rated health**	**Self-rated health**	**Self-rated health**	**Number of chronic diseases**	**Number of chronic diseases**	**Number of chronic diseases**
Endowment insurance	0.013	0.073*	0.029	−0.038	−0.005	−0.059
	(0.064)	(0.039)	(0.040)	(0.057)	(0.035)	(0.037)
Medical insurance	−0.184***	−0.025	−0.049	0.371***	0.200***	0.102**
	(0.069)	(0.049)	(0.049)	(0.062)	(0.045)	(0.044)
Other social assistance	0.078	0.034	0.027	−0.127*	−0.043	−0.119**
	(0.098)	(0.060)	(0.060)	(0.076)	(0.0476)	(0.047)
Endowment insurance × infrastructure	0.006			0.004		
	(0.011)			(0.010)		
Medical insurance × infrastructure	−0.041***			−0.049***		
	(0.010)			(0.009)		
Other social assistance × infrastructure	−0.010			0.012		
	(0.017)			(0.013)		
Endowment insurance × activity places		−0.008			−0.003	
		(0.005)			(0.003)	
Medical insurance × activity places		−0.006*			−0.009***	
		(0.003)			(0.003)	
Other social assistance × activity places		0.001			−0.010	
		(0.005)			(0.007)	
Endowment insurance × medical institutions			−0.012			−0.041
			(0.016)			(0.035)
Medical insurance × medical institutions			0.014			0.018
			(0.014)			(0.013)
Other social assistance × medical institutions			0.001			−0.005
			(0.025)			
**Control variables**	**Control**	**Control**	**Control**	**Control**	**Control**	**Control**
*N*	4,845	4,845	4,845	9,785	9,785	9,785
*R* ^2^	0.013	0.014	0.013	0.091	0.093	0.093

* *p <0.1,*

** *p <0.05, and*

*** *p <0.01*.

**Table 4:** Impact of formal social support on mental health of older adults (regulatory effect of community environment).

	**(1)**	**(2)**	**(3)**	**(4)**	**(5)**	**(6)**
	**Degree of depression**	**Degree of depression**	**Degree of depression**	**Cognitive level**	**Cognitive level**	**Cognitive level**
Endowment insurance	0.079	0.039	0.070**	0.095	−0.033	−0.010
	(0.053)	(0.032)	(0.033)	(0.063)	(0.038)	(0.039)
Medical insurance	0.072	0.016	0.074*	0.091	0.119**	0.292***
	(0.058)	(0.041)	(0.041)	(0.069)	(0.048)	(0.048)
Other social assistance	−0.576***	−0.469***	−0.565***	−1.144***	−1.019***	−1.328***
	(0.071)	(0.043)	(0.043)	(0.084)	(0.051)	(0.050)
Endowment insurance × infrastructure	−0.001			0.031***		
	(0.009)			(0.011)		
Medical insurance × infrastructure	0.028***			0.054***		
	(0.008)			(0.010)		
Other social assistance × infrastructure	0.015			0.006		
	(0.012)			(0.0152)		
Endowment insurance × activity places		0.007**			0.015***	
		(0.003)			(0.004)	
Medical insurance × activity places		0.007**			0.022***	
		(0.003)			(0.003)	
Other social assistance × activity places		−0.019***			−0.048***	
		(0.004)			(0.005)	
Endowment insurance × medical institutions			0.015			0.052***
			(0.014)			(0.016)
Medical insurance × medical institutions			−0.001			0.004
			(0.012)			(0.014)
Other social assistance × medical institutions			−0.030*			−0.039*
			(0.017)			(0.020)
**Control variables**	**Control**	**Control**	**Control**	**Control**	**Control**	**Control**
_cons	0.108	−0.030	−0.049	3.769***	3.572***	3.508***
	(0.131)	(0.124)	(0.123)	(0.158)	(0.145)	(0.145)
*N*	9,785	9,785	9,785	9,785	9,785	9,785
*R* ^2^	0.015	0.016	0.015	0.182	0.213	0.205

* *p <0.1,*

** *p <0.05, and*

*** *p <0.01*.

**Table 5:** Impact of informal social support on physical health of older adults (regulatory effect of community environment).

	**(1)**	**(2)**	**(3)**	**(4)**	**(5)**	**(6)**
	**Self-rated health**	**Self-rated health**	**Self-rated health**	**Number of chronic diseases**	**Number of chronic diseases**	**Number of chronic diseases**
The number of surviving children	0.0208	0.045***	0.043***	0.038*	0.036**	0.037**
	(0.0230)	(0.015)	(0.016)	(0.020)	(0.014)	(0.014)
Number of siblings	0.0615***	0.023**	−0.002	0.074***	0.055***	0.041***
	(0.0159)	(0.010)	(0.010)	(0.014)	(0.009)	(0.009)
Children's financial support	−0.0000112**	−0.053**	0.009	0.077**	0.022	0.021
	(0.00000547)	(0.024)	(0.025)	(0.036)	(0.019)	(0.018)
Parental financial support	0.0000683	−0.131	−0.071	0.254	−0.096	−0.104
	(0.0000654)	(0.105)	(0.095)	(0.467)	(0.139)	(0.125)
The number of surviving children × infrastructure	0.00388			−0.0003		
	(0.00390)			(0.003)		
The number of siblings × infrastructure	−0.0119***			−0.009***		
	(0.00279)			(0.003)		
Children's financial support × infrastructure	0.00000186**			−0.013**		
	(0.000000912)			(0.006)		
Parental financial support × infrastructure	−0.0000197			−0.093		
	(0.0000162)			(0.112)		
The number of surviving children × activity places		−0.0003			0.0003	
		(0.0013)			(0.001)	
The number of siblings × activity places		−0.0022**			−0.003***	
		(0.0009)			(0.001)	
Children's financial support × activity places		0.002**			−0.001	
		(0.001)			−0.001	
Parental financial support × activity places		0.028			−0.006	
		(0.019)			(0.014)	
The number of surviving children × medical institutions			0.001			0.002
			(0.006)			(0.005)
The number of siblings × medical institutions			0.003			−0.005
			(0.004)			(0.004)
Children's financial support × medical institutions			−0.005			−0.011
			(0.013)			(0.009)
Parental financial support × medical institutions			0.114			−0.033
			(0.141)			
**Control variables**	**Control**	**Control**	**Control**	**Control**	**Control**	**Control**
*N*	4,545	4,545	4,545	9,785	9,785	9,785
*R* ^2^	0.017	0.018	0.017	0.090	0.090	0.089

* *p <0.1,*

** *p <0.05, and*

*** *p <0.01*.

**Table 6:** Impact of informal social support on mental health of older adults (regulatory effect of community environment).

	**(1)**	**(2)**	**(3)**	**(4)**	**(5)**	**(6)**
	**Degree of depression**	**Degree of depression**	**Degree of depression**	**Cognitive level**	**Cognitive level**	**Cognitive level**
The number of surviving children	−0.035*	−0.040***	−0.043***	−0.114***	−0.097***	−0.077***
	(0.018)	(0.013)	(0.0131)	(0.021)	(0.014)	(0.014)
The number of siblings	−0.034**	−0.011	0.0063	−0.015	0.007	0.028***
	(0.013)	(0.009)	(0.008)	(0.014)	(0.009)	(0.009)
Children's financial support	−0.044	−0.024	−0.012	0.065*	0.061***	0.016
	(0.035)	(0.022)	(0.018)	(0.037)	(0.019)	(0.018)
Parental financial support	−0.563	−0.300	−0.117	−0.759	−0.051	−0.077
	(0.532)	(0.281)	(0.144)	(0.478)	(0.136)	(0.123)
The number of surviving children × infrastructure	0.0001			0.016***		
	(0.003)			(0.0035)		
The number of siblings × infrastructure	0.008***			0.007***		
	(0.002)			(0.003)		
Children's financial support × infrastructure	0.012*			−0.011*		
	(0.007)			(0.006)		
Parental financial support × infrastructure	0.121			0.176		
	(0.130)			(0.115)		
The number of surviving children × activity places		−0.001			0.005***	
		(0.001)			(0.001)	
The number of siblings × activity places		0.003***			0.003***	
		(0.001)			(0.001)	
Children's financial support × activity places		0.005*			−0.003***	
		(0.003)			(0.0008)	
Parental financial support × activity places		0.046			0.006	
		(0.036)			(0.014)	
The number of surviving children × medical institutions			−0.002			0.008
			(0.004)			(0.005)
The number of siblings × medical institutions			0.001			0.002
			(0.003)			(0.003)
Children's financial support × medical institutions			0.016*			−0.008
			(0.009)			(0.008)
Parental financial support × medical institutions			0.096			0.0717
			(0.087)			(0.074)
**Control variables**	**Control**	**Control**	**Control**	**Control**	**Control**	**Control**
	−0.140	−0.305**	−0.333**	3.516***	3.235***	3.162***
	(0.155)	(0.142)	(0.141)	(0.174)	(0.155)	(0.154)
*N*	9,785	9,785	9,785	9,785	9,785	9,785
*R* ^2^	0.019	0.013	0.018	0.207	0.228	0.223

* *p <0.1,*

** *p <0.05, and*

*** *p <0.01*.

In Table 3, Columns (1), (2), and (3) report the impact of formal social support on self-rated health, as well as show the results for the interaction terms of formal social support with the infrastructure index, activity place index, and medical institutions index. The interaction terms among the infrastructure index, activity place index, endowment insurance, and other social assistance programs were not significant; however, the interaction terms among these two indexes and medical insurance were significant, with a negative coefficient. This might be because a community with better infrastructure and activity places enables older adults to communicate more among themselves, increasing their self-rated health; further, it may be that older adults with medical insurance are more likely to be in a poor physical condition, which may lead to worse self-rated health. The interaction term of the medical institutions' index with endowment insurance, with medical insurance, and with other social assistance programs, was not significant.

Columns (4), (5), and (6) show that the interaction terms of the infrastructure index and activity place index with endowment insurance and other social assistance programs were not significant, but the interaction items with medical insurance are significant and the coefficient is negative. This might be because communities with better infrastructure and activity places indicate older adults have higher living standards, and that they may have access to higher-quality medical insurance, which may be used more actively and reduce the number of chronic diseases. The interaction between the index of medical institutions and endowment insurance, medical insurance, and other social assistance programs, was not significant.

Columns (1), (2), and (3) in Table 4 show results related to the degree of depression and formal social support. The interactions between the infrastructure index and endowment insurance and other social assistance programs were not significant; however, the interaction between infrastructure index and medical insurance was significant and positive. This may be because communities with better infrastructure may enable older adults to present a generally more positive physical and mental health status, reducing the probability of depression. The interactions among activity place index and endowment insurance, medical insurance, and other social assistance programs were all significant; the first two coefficients were positive and the third was negative. It may be that in communities with better activity places, older adults with a pension or medical insurance may present a generally more positive physical and mental health status, reducing the probability of depression. Meanwhile, communities with better activity places that provide access to other social assistance programs may enable older adults who receive such alternative programs to experience discrimination, increasing the probability of depression. Furthermore, the interaction between the medical institutions index and other social assistance programs was significant and negative. The possible reasons may be similar to those described for the activity place index. Meanwhile, the interactions among medical institutions index and endowment insurance, and medical insurance, were not significant.

Columns (4), (5), and (6) show the results regarding cognitive level and formal social support. The interaction between infrastructure index and other social assistance programs was not significant; however, it was significant and positive for endowment insurance and medical insurance. This might be because in communities with better infrastructure, older adults tend to have generally more positive physical and mental health status, leading to a higher cognitive level. Moreover, the interactions among activity place index and endowment insurance, medical insurance, and other social assistance programs were all significant, with the first two being positive and the third being negative. Additionally, the interaction between medical institutions index and medical insurance was not significant, but those among medical institutions index and endowment insurance and other social assistance programs, were significant. Specifically, the former coefficient was positive and the latter coefficient was negative. The possible causes are similar to those already described.

Columns (1), (2), and (3) in Table 5 show the results pertaining to self-rated health and informal social support. The interaction between the infrastructure index and the number of children, and between the first and parental financial support, were non-significant; however, the interaction between the infrastructure index and children's financial support, and the number of siblings, were both significant: the coefficient of the former was positive and of the latter was negative. The interaction between the activity place index and both the number of children and parental financial support were non-significant. The interactions between activity place index and both children's financial support and the number of siblings were significant: the coefficient of the former was positive and of the latter was negative. These may have been significant because, communities with better infrastructure and activity places may facilitate older adults' access to their children's financial support, which may help improve older adults' physical health. Second, such communities may facilitate the residency of more siblings of older adults, and sibling affairs (which may be of a trivial nature) may worsen older adults' physical health. Meanwhile, the interaction among the medical institutions' index, the number of children, children's financial support, parental financial support, and the number of siblings, were not significant.

Columns (4), (5), and (6) in Table 5 pertain to the results of the number of chronic diseases and informal social support. The interactions between infrastructure index and the number of children and parental financial support were not significant; the interactions between infrastructure index and children's financial support and the number of siblings, were significant and negative. The possible reasons are similar to those for self-rated health. The interactions among activity place index, medical institutions index, the number of children, children's financial support, and parental financial support were all non-significant.

Columns (1), (2), and (3) in Table 6 pertain to the degree of depression and informal social support. The interactions between the infrastructure index and the number of children and parental financial support, were not significant; however, the interactions between infrastructure index and the number of siblings and children's financial support were significant and positive. The interactions between activity place index and the number of children and parental financial support were not significant. The interactions between activity place index and the number of siblings and children's financial support were significant and positive. Additionally, the interactions between medical institutions index and the number of children and parental financial support were not significant; however, the interaction term between medical institutions index and children's financial support was significant and positive.

Columns (4), (5), and (6) in Table 6 pertain to cognitive level and informal social support. The interaction between infrastructure index and parental financial support was not significant; however, the interactions among infrastructure index and the number of children, the number of siblings, and children's financial support were significant, with the first two being positive and the third being negative. The interaction between activity place index and parental financial support was not significant. However, the interactions among activity place index and the number of children, the number of siblings, and children's financial support were all significant; the first two were positive and the third was negative. These results may be related to changes in psychological pressure among older adults after they receive informal social support in different community environments.

The result could be supported by the research of “Everard et al. (50).”

## Conclusions

Using data from the 2011 and 2015 waves of the CHARLS, we discussed the relationship between social support and older adults' health in different community environments. The results showed that both formal and informal social support significantly affected both physical (i.e., self-rated health and the number of chronic diseases) and mental health (i.e., degree of depression and cognitive level). The main manifestations were as follows: the greater the endowment insurance and the other social assistance programs, the better the physical health; further, no medical insurance and children's economic support also led to better physical health. Additionally, having endowment insurance and medical insurance, but no other social assistance programs; having more siblings and children's financial support; and having a lower number of living children; all led to better mental health.

Results showed that social support affects older adults' health through health behavior and satisfaction. Specifically, the moderating role of community environment mostly manifested as a substitution: the higher the community environmental index, the lower the impact of social support on older adults' health. Therefore, to build a reasonable and effective social support system for Chinese older adults and improve their health, the following suggested:

Stakeholders should strengthen formal social support sources for older adults, optimizing pension policies and reasonably supplementing commercial pension insurances. In the face of the pension gap, which is a major concern nationwide, we can adopt methods such as delaying retirement, developing supplementary pension insurances, state-owned asset allocations, pension integration, improving pension investment scale and yield, and issuing government bonds to ensure a better economic life for older adults. Furthermore, commercial endowment insurance may be an effective supplement to the basic endowment insurance policy. The development of China's commercial old-age insurance is in its primary stage, therefore efforts should be made to “improve the multi-layer social security system to cover the whole population, to coordinate urban and rural areas, to be fair, unified, and sustainable.”

Stakeholders should improve the national health insurance system and give full play to the role of social assistance. They should also promote constructing a health system for older adults and strengthen the health management of chronic diseases; this entails accelerating the implementation of a comprehensive prevention and control strategy for dealing with chronic diseases. Moreover, stakeholders should also strengthen the construction of urban and rural social assistance systems, give full play to the role of bottom-up security, and promote and rely on charities to organize fund-raising and related activities for urban and rural older adults in extreme poverty; these actions may enable the role of the national social assistance system to be utilized to its fullest capacity.

Those invested in this topic should endeavor to improve the informal social support of Chinese older adults, carry forward the tradition of respecting and serving older adults, and implement strategies that assist in promoting self-help and mutual assistance in this population. Deeply influenced by traditional Confucianism, China has formed a traditional model of “family pension,” where children support their older adult parents. Hence, children should fulfill their responsibilities of supporting their older adult parents financially, with healthcare-related topics, and spiritually. In addition, it may be possible to implement strategies where older adults provide various types of support for each other, such as through “pair assistance” or a “time bank” model.

Finally, interested parties should enable the moderating role of community environment on the relationship between social support and health to be fully exerted, as well as build a community-based pension system. By improving the community environment, promoting more cultural and recreational activities, and improving its infrastructure, we may be able to increase the impact of this moderating effect, ultimately impacting older adults' health.

This article has several limitations. The study only intercepts cross-sectional data of a certain year for analysis, without considering the dynamic loss of health levels. In addition, health levels can be changed through subjective behavior. The study does not fully consider the intermediary effect of subjective behavior; that is, it has not investigated the impact of social support factors on later subjective initiatives and further effects on health levels. Future research should add the tracking data of the following years to the next research, establish a complete panel, and conduct a comprehensive and dynamic investigation on the samples. It should also add the intermediary factor of health behavior into the influence mechanism to further investigate how social support factors affect health level.

## Data Availability Statement

The datasets presented in this study can be found in online repositories. The names of the repository/repositories and accession number(s) can be found below: http://charls.pku.edu.cn/.

## Author Contributions

DL: conceptualization, formal analysis, funding acquisition, and investigation. XL: data curation, methodology, and writing—original draft preparation. YZ: supervision, visualization, and writing—review and editing. All authors contributed to the article and approved the submitted version.

## Funding

This study was supported by the Fundamental Research Funds for the Central Universities, Zhongnan University of Economics and Law (grant number: 2722021BZ034), the Major Project of Philosophy and Social Sciences in Hubei Province (Project No. 21ZD011), and the Research on Humanities and Social Sciences of Hubei Education Department (grant number: 21G027).

## Conflict of Interest

The authors declare that the research was conducted in the absence of any commercial or financial relationships that could be construed as a potential conflict of interest.

## Publisher's Note

All claims expressed in this article are solely those of the authors and do not necessarily represent those of their affiliated organizations, or those of the publisher, the editors and the reviewers. Any product that may be evaluated in this article, or claim that may be made by its manufacturer, is not guaranteed or endorsed by the publisher.
