# Supplementary material for: The Moderating Effect of Community Environment on the Association Between Social Support and Chinese Older Adults' Health: An Empirical Analysis Study
Source: Front Public Health. 2022 Apr 27;10:855310. doi: 10.3389/fpubh.2022.855310 (PMC9092342; doi:10.3389/fpubh.2022.855310)
Supplement: Supplementary file 1 [file Table_1.DOCX]

**Appendix: The Table of Research variables**

| Variables | Name | | | Code in CHARLS | Type | Measurement standard |
| --- | --- | --- | --- | --- | --- | --- |
| **Explained**  **variable** | Physical health | self-rated health | | DA001 | Categorical | Very good=5, Good =4, Fair =3, Poor =2,Very poor=1 |
|  |  | the number of chronic diseases | | DA007 | Continuous | The chronic diseases in the CHARLS data include Hypertension、Dyslipidemia、Diabetes、Cancer、Chronic lung diseases、Liver disease、Heart disease、Stroke、Kidney disease、Stomach disease、Psychiatric problems、Memory-related disease、Arthritis 、Asthma. Range: 0–14. |
|  | Mental health | degree of depression | | DC009-DC018 | Categorical | After assessing participants’ total scores, we re-coded the data, assigning participants to two groups based on their scores: 1 (no depression) for those with a score of 20 and below, and 0 (depression) for those with scores over 20. |
|  |  | cognitive level | | DC001-DC003 | Continuous | Participants responded to items on year, month, day, week and season. One point was assigned to each correct answer. One point was assigned to each correct answer.Range:0-5 |
| ***Explanatory***  ***variables*** | Formal social support | endowment insurance | | FN002_w3/FN069_w3 | Categorical | 1 have，0 no |
|  |  | medical insurance | | EA001 | Categorical | 1 have，0 no |
|  |  | other social assistance | | GA003 | Categorical | 1 have，0 no |
|  | Informal social support | Number of surviving children | | CB050_w3 | Continuous | Range:0-15 |
|  |  | Number of siblings | | CC000_W3_1 | Continuous | Range:0-14 |
|  |  | Parental financial support | | CE002 | Continuous | Range:0-6000 |
|  |  | Children's financial support | | CE009 | Continuous | Range:5-328000 |
| **Control variables** | sex | | | A005_w3 | Categorical | 1 male，0 female |
|  | age | | | BA002 | Continuous |  |
|  | residency | | | BB000_W3_2 | Categorical | 1 urban，0 rural areas |
|  | marital status | | married | BE001 | Categorical | 1 married，0 other marital status |
|  |  |  | separated | BE001 | Categorical | 1 separated，0 other marital status |
|  |  |  | divorced | BE001 | Categorical | 1 divorced，0 other marital status |
|  |  |  | widowed | BE001 | Categorical | 1 widowed，0 other marital status |
|  |  |  | cohabitated | BE001 | Categorical | 1 cohabitated，0 other marital status |
|  |  |  | single | BE001 | Categorical | 1 single，0 other marital status |
